# Consumption of Artificially-Sweetened Soft Drinks in Pregnancy and Risk of Child Asthma and Allergic Rhinitis

**DOI:** 10.1371/journal.pone.0057261

**Published:** 2013-02-27

**Authors:** Ekaterina Maslova, Marin Strøm, Sjurdur F. Olsen, Thorhallur I. Halldorsson

**Affiliations:** 1 Centre for Fetal Programming, Department of Epidemiology, Statens Serum Institut, Copenhagen, Denmark; 2 Department of Nutrition, Harvard School of Public Health, Boston, Massachusetts, United States of America; 3 Faculty of Food Science and Nutrition, School of Health Sciences, University of Iceland, Reykjavik, Iceland; 4 Unit for Nutrition Research, Landspitali University Hospital, Reykjavik, Iceland; Ludwig-Maximilians-University Munich, Germany

## Abstract

**Background:**

Past evidence has suggested a role of artificial sweeteners in allergic disease; yet, the evidence has been inconsistent and unclear.

**Objective:**

To examine relation of intake of artificially-sweetened beverages during pregnancy with child asthma and allergic rhinitis at 18 months and 7 years.

**Methods:**

We analyzed data from 60,466 women enrolled during pregnancy in the prospective longitudinal Danish National Birth Cohort between 1996 and 2003. At the 25th week of gestation we administered a validated Food Frequency Questionnaire which asked in detail about intake of artificially-sweetened soft drinks. At 18 months, we evaluated child asthma using interview data. We also assessed asthma and allergic rhinitis through a questionnaire at age 7 and by using national registries. Current asthma was defined as self-reported asthma diagnosis and wheeze in the past 12 months. We examined the relation between intake of artificially-sweetened soft drinks and child allergic disease outcomes and present here odds ratios with 95% CI comparing daily vs. no intake.

**Results:**

At 18 months, we found that mothers who consumed more artificially-sweetened non-carbonated soft drinks were 1.23 (95% CI: 1.13, 1.33) times more likely to report a child asthma diagnosis compared to non-consumers. Similar results were found for child wheeze. Consumers of artificially-sweetened carbonated drinks were more likely to have a child asthma diagnosis in the patient (1.30, 95% CI: 1.01, 1.66) and medication (1.13, 95% CI: 0.98, 1.29) registry, as well as self-reported allergic rhinitis (1.31, 95% CI: 0.98, 1.74) during the first 7 years of follow-up. We found no associations for sugar-sweetened soft drinks.

**Conclusion:**

Carbonated artificially-sweetened soft drinks were associated with registry-based asthma and self-reported allergic rhinitis, while early childhood outcomes were related to non-carbonated soft drinks. These results suggest that consumption of artificially-sweetened soft drinks during pregnancy may play a role in offspring allergic disease development.

## Introduction

It has been hypothesized that diet during pregnancy may modulate child immune system development and later allergic disease. Past studies have examined numerous dietary factors during pregnancy, including fruits and vegetables [1]–[6], fish [1]–[5], [7]–[12] and fish oil [13], butter and margarine [2], [3], [5], [7], nut and peanuts [2], [4], [14], and dairy and milk intake [2]–[5], [14], [15] in relation to allergic disease in the child. We recently conducted an analysis of the relation between milk and yoghurt consumption during pregnancy and wheeze, asthma, and allergic rhinitis in the Danish National Birth Cohort (DNBC) [16]. We found that the strongest and most consistent association was between maternal low-fat yoghurt intake and child asthma and allergic rhinitis. These results suggested a causal agent specific to low-fat yoghurt. We hypothesized that artificial sweeteners could be a plausible candidate as these compounds are often added to low-fat/’light’ products to compensate for flavour or reduce sugar content [17]. The current state of knowledge on the role of artificial sweeteners in allergic disease development is at best limited. A few studies have examined aspartame in relation to inflammation; yet, these results have been inconsistent [18]–[20]. A study from the 1980s [21] examined *in vitro* immunological properties and found small inhibitory effects on antigen-induced histamine release from cultured mouse mast cells. However, the authors could not explain the origin or meaning of this effect. Other studies have shown improvements in dermatitis with aspartame avoidance [22], [23], while yet others found no elicit allergic reactions [24], [25]. This suggests that improvement in dermatitis is most likely a relatively rare side effect of aspartame avoidance. To our knowledge, there are no epidemiological studies assessing the relation between intake of artificial sweeteners during pregnancy and asthma or other allergic diseases.

We therefore decided to examine whether artificially-sweetened soft drinks during pregnancy modify offspring risk of wheeze, asthma, and allergic rhinitis in the DNBC. We chose soft drink as the exposure because these have been identified as a primary source of artificial sweeteners [26]. We included intake of sugar-sweetened beverages as a control for potential activity of other nutritional components and additives in soft drinks [26].

## Methods

### Ethics Statement

Mothers provided written informed consent on behalf of their children. The Regional Scientific Ethics Committee for the municipalities of Copenhagen and Frederiksberg approved all study protocols, and all procedures were in accordance with the Declaration of Helsinki.

### Population and Study Design

Participants in the DNBC, a large prospective cohort study, were enrolled between January 1996 and October 2002 during their first antenatal visit between weeks 6 and 10 of gestation [27]. All women living in Denmark were eligible if they could speak Danish and were planning to carry to term. About 60% of all eligible women received an invitation from their general practitioner; of those 60% chose to enrol. Women participated in four telephone interviews, twice during pregnancy and twice postpartum, at 6 and 18 months. They also filled out a food frequency questionnaire (FFQ) around the 25^th^ week of gestation. We have followed the mother-child pairs through national registries, linked by the unique identity number given to all Danish citizens. A questionnaire regarding the child was sent to the mothers when the child was 7 years.

A total of 101,045 pregnancies were enrolled into the study. To avoid dependency among correlated observations, we included only the first liveborn singleton child enrolled for each woman (n = 89,333). We further excluded multiple births (n = 87,056). After eliminating women with missing exposure data, 60,466 women had information on artificially-sweetened carbonated beverage consumption.

### Exposure Variables

Maternal diet assessment was based on a validated 360-item semi-quantitative FFQ completed around gestation week 25 and covered intake during the previous four weeks [28]. Intake of individual food items were estimated in grams per day based on standard portion sizes; total energy and nutrients were quantified using Danish food tables [29]. Women were asked a number of questions on beverage consumption with response categories ranging from never to > = 8 servings/d. One serving was equivalent to one glass. We used four beverage items in our analyses: carbonated soft drinks/cola (sugar sweetened), carbonated soft drinks/cola (sugar-free, light), noncarbonated soft drinks (sugar sweetened), and noncarbonated soft drinks (sugar-free, light). The wording of the responses used the words sugar-free and light to refer to products that contained artificial sweeteners. We distinguished between carbonated and non-carbonated beverages as they contain different types of artificial sweeteners [26]. These beverages were subject to a repeatability study where 103 women filled in the FFQ twice during pregnancy (in week 25 and 35 of gestation). For these women the observed Spearman correlation coefficient for all four types of soft drink consumption was around 0.7 [30].

### Outcome Measurements

#### Asthma at 18 months

Women were telephone interviewed when the child was 18 months (N∼45,000). The women answered questions about child health and diagnoses since birth. We defined *asthma at 18 months* as self-reported doctor asthma diagnosis.

#### Asthma and allergic rhinitis at 7 years

We evaluated asthma and allergic rhinitis diagnosis at the 7 year follow-up by numerous sources. *Current asthma at age 7* (N∼38,000) by self-report was defined as a positive response to standardized International Study of Asthma and Allergies in Childhood questions on doctor-diagnosed asthma and wheezing symptoms in the past 12 months [31], [32]. *Ever allergic rhinitis* (N∼38,000) by self-report was based on a reported doctor-diagnosis of hay fever.

We further used data on hospital admissions collected by the mandatory Danish National Patient Registry (DNPR). Admission information has been collected since 1977, and emergency room and outpatient contacts since 1995. The registry has been validated against asthma diagnosis from hospital records [33]. We extracted data from the DNPR in Aug 2010 and linked it to our data using the CPR number. We identified *ever admitted asthma* (N∼38,000) cases using the International Classification of Disease 10 (ICD-10) for asthma (J45, J45.0, J45.1, J45.8, J45.9, J46, and J46.9).

We also used the Register of Medicinal Product Statistics (RMPS) that was initiated in 1995 and contains detailed individual-level dispensary information [34]. Based on a previous validation study [35] we combined Anatomical Therapeutic Chemical codes for the classification of *ever medication-related asthma* (N∼38,000) cases. The outcome was defined as at least two prescriptions of drugs for obstructive airway disease, except for beta-2-agonists as liquid, inhaled beta-2-agonists only once, or inhaled steroid only once.

Both registry outcomes were limited to children with self-reported data to avoid discordance in findings due to differences in study populations.

### Covariates

We collected information of a number of covariates. These included socioeconomic status by parental education level and occupation (SES: high level proficiency, medium level proficiency, skilled, unskilled, student, unemployment), maternal age at birth of child (< = 20 years, 21–39 years, > = 40 years), parity (nulli- and multiparous), maternal prepregnancy Body Mass Index (BMI in kg/m^2^) (< = 18.5, 18.6–24.9, 25.0–29.9, 30–34.9, > = 35.0), maternal smoking during pregnancy (nonsmoker, occasional smoker, current smoker), maternal exercise during pregnancy (yes/no), gestational weight gain (g/week), breastfeeding duration (none, 0–1, 2–3, 4–6, 7–9, > = 10 months), birth weight (in grams), gestational age (in days since the last menstrual period), child sex, maternal and paternal history of asthma and allergies, and total energy intake. We also examined, in stability analyses, dietary variables (fruits, vegetables, alcohol, vitamin A, D, E, selenium, and zinc intake from diet and supplements); and mode of delivery (cesarean section, vaginal), pets at home, exposure do smoking during infancy (no, occasional, yes), daycare attendance during infancy, and use of antibiotics and paracetamol during pregnancy and child use of antibiotics (against ear infections) at 6 month.

### Statistical Analysis

We evaluated the distribution of covariates across categories of beverage intake for potential confounding factors. We age-standardized the distributions using direct standardization as there was a significant difference in intake across maternal age categories. Final set of covariates were determined by chi-square and partial F-tests with a P<0.10 as well as *a priori* considerations based on the current literature. We excluded covariates suspected to be intermediates on the causal pathways, such as birth weight, gestational age, and gestational weight gain, to avoid over-adjusting the model. The importance of these variables was instead assessed using stratification. In the final logistic regression model, we adjusted for maternal age, smoking, parity, prepregnancy BMI, physical activity, breastfeeding duration, socio-economic status, child sex, maternal history of asthma, maternal history of allergies, paternal history of asthma, paternal history of allergies, and energy (in quintiles). Soft drinks intake was entered as an indicator variable and individual exposure categories compared to the lowest category. We collapsed categories at the higher end due to scarce data on high-consumers and to increase power. We therefore ended up with four intake categories (never, <1serving/week, weekly (2–6 times/week), > = 1serving/day). When comparing results to the non-collapsed variables, there was a slight change in effect estimates and confidence intervals, but directionality remained the same. We estimated odds ratios (ORs) and 95% CI for the final models. Only exposures with strongest and most consistent results are presented in the main text; the reader is referred to the **Supporting Materials** for additional details. Median values (0, 0.5, 3.5, and 7) for the intake categories were modelled as a continuous variable to evaluate P-value for trend. We evaluated the independent associations for each outcome by entering all beverage variables into the same model. We also collapsed intake of artificially-sweetened beverage (carbonated and non-carbonated) to assess the full effect of the sweeteners. Since the same frequency number could be obtained across two different individual intake categories we ended up with the following combined categories (‘never to <1serv/week’, ‘<1serv/week’, ‘<1serv/week to weekly’, ‘weekly to > = 1serv/day’).

All tests were two-sided and statistical significance was considered at P<0.05. The analyses were performed using the Statistical Analyses System software (release 9.2; SAS Institute, Cary, NC).

## Results

### Study Cohort

A total of 60,466 women had information on artificially-sweetened carbonated beverage intake. Majority of women were between the ages 21 and 39 years (98%), of higher socioeconomic position (high level proficiencies: 23%), and nulliparous (53%) (**Table 1**). More than 68% of all women reported a prepregnancy BMI within 18.6–24.9 kg/m^2^. Nearly 25% of participants reported having smoked during pregnancy with 12% being daily smokers. Prevalence of maternal history of asthma and allergies was 9% and 32% respectively. Among the exposure variables, intake of sugar-sweetened non-carbonated soft drinks was highest with 22% consuming > = 1 servings/day, followed by artificially-sweetened non-carbonated soft drinks at 13%.

**Table 1:** Age-standardized covariate distribution across categories of maternal consumption of artificially-sweetened carbonated soft drinks during pregnancy, N = 60,465.

Frequency of intake	N^1^ (%)	Never N = 40,523%or means (SD)	<1 serv/week N = 7,588%or means (SD)	Weekly N = 9,971%or means (SD)	> = 1 serv/day N = 2,383%or means (SD)
**Maternal age** (years)*					
≤20	586 (1)	1	1	1	1
21–39	59,311 (98)	98	98	98	98
≥40	567 (1)	1	1	1	1
**Prepregnancy BMI** (kg/m^2^)					
≤18.5	2,476 (4)	5	3	2	2
18.6–24.9	38,212 (68)	71	68	61	54
25.0–29.9	10,885 (19)	18	20	24	27
30.0–34.9	3,299 (6)	5	6	9	11
≥35.0	1,219 (2)	2	3	3	5
**Physical activity**					
Yes	22,399 (39)	37	43	41	37
**Smoking in pregnancy**					
Current smokers	7,470 (12)	13	9	10	17
**Parity**					
Nullipara	30,590 (53)	51	58	56	52
**Socioeconomic position**					
High level proficiencies	12,449 (23)	23	23	22	22
Medium level proficiencies	16,692 (31)	30	32	32	30
Skilled	14,601 (27)	26	27	28	29
Unskilled	6,810 (13)	13	12	12	14
Students	2,425 (4)	5	4	4	4
Unemployed	1,462 (3)	3	2	2	2
**Breastfeeding duration**					
No breastfeeding	862 (2)	2	2	2	4
0–1 months	3,988 (9)	9	8	11	13
2–3 months	4,424 (10)	10	9	11	14
4–6 months	8,004 (19)	18	18	20	21
7–9 months	11,636 (27)	27	29	28	22
≥10 months	14,078 (33)	34	33	28	26
**Maternal asthma**					
Yes	5,289 (9)	9	9	9	10
**Maternal allergies**					
Yes	18,404 (32)	32	31	32	33
**Paternal asthma**					
Yes	4,720 (8)	8	8	9	9
**Paternal allergies**					
Yes	13,739 (24)	24	24	24	24
**Child gender**					
Male	30,754 (51)	51	52	51	52
**Gestational weight gain (g/week)**	41,681	468 (213)	465 (211)	465 (223)	460 (241)
**Birth weight (g)**	57,403	3,572 (571)	3,593 (573)	3,596 (577)	3,595 (588)
**Gestational age (days)**	60,454	280 (14)	280 (15)	280 (13)	279 (16)
**Dietary intake**					
Energy intake (kJ/day)	60,227	10,208 (2,706)	9,707 (2,570)	9,787 (2,588)	9,945 (2,809)
Fruit intake (g/day)	60,461	320 (267)	327 (270)	329 (258)	335 (327)
Vegetable intake (g/day)	60,461	129 (99)	130 (95)	128 (96)	132 (129)
Alcohol (g/day)	60,227	1.5 (2.3)	1.6 (2.1)	1.7 (2.3)	1.7 (2.8)
Total vitamin A (RE/day)	60,227	1,500 (24,090)	1,297 (569)	2,125 (50,352)	2,672 (68,549)
Total vitamin D (µg/day)	60,227	8.9 (5.5)	9.2 (5.5)	9.2 (5.6)	8.9 (5.6)
Total vitamin E (α-TE/day)	60,227	16.4 (19.7)	16.3 (19.9)	15.8 (15.6)	15.4 (14.7)
Total selenium (µg/day)	60,227	70.1 (27.9)	72.8 (27.4)	72.6 (26.9)	70.9 (26.6)
Total zinc (mg/day)	60,227	19.6 (7.7)	20.2 (7.7)	20.1 (7.4)	19.7 (7.4)

Values are standardized to the age distribution of the study population.

* Value not age-adjusted.

1 Total may be <N due to missing values.

When comparing women with and without self-reported child asthma data at 18 months and 7 years we found that, at 18 months, the 45,604 participants (vs. 16,269 non-participants) in the analyses were of higher sociodemographic status (54% vs. 51%), were multiparous (48% vs. 44%), did not smoke during pregnancy (76% vs. 72%), and had slightly lower gestational weight gain (464 vs. 475 g/week). At 7 years, compared to women without data on current child asthma (N = 22,947), the 38,926 women with data on child asthma were of higher sociodemographic status (55% vs. 50%), were more like to have a BMI within 18.6–24.9 kg/m^2^ (70% vs. 65%), exercise (40% vs. 36%), and not smoke during pregnancy (77% vs. 72%). No differences were found for maternal age, parental asthma and allergies, or intake of selected micronutrients during pregnancy.

### Exposure Associations

We assessed intake of artificially-sweetened carbonated beverages across age-standardized study participant characteristics (**Table 1**). Compared to never consumers, women with daily intake of artificially-sweetened carbonated soda drinks tended to be of a skilled and unskilled level proficiency, have lower parity, and a prepregnancy BMI > = 25 kg/m^2^. They also smoked more during pregnancy and breastfed <7 months. They reported lower energy intake and higher intake of fruit, vegetables, and alcohol, but lower intake of vitamin E. Trends were similar for artificially-sweetened non-carbonated beverages.

### Multivariate Analysis

#### Child asthma at 18 months follow-up

The prevalence of child asthma was 17% (N = 7,628/44,638). At the 18 month follow-up, only intake of artificially-sweetened non-carbonated soft drinks were associated with higher risk of self-reported child asthma diagnosis (> = 1serving/day vs. never: 1.23, 95% CI: 1.13, 1.33) (**Table** **2**). No associations were found for sugar-sweetened beverages (**Tables S1–2**). Combining intake of artificially-sweetened beverages generated effect estimates between the two sub-categories and was strongest for child asthma (> = 1serving/day vs. never: 1.14, 95% CI: 1.00, 1.28) (data not shown).

**Table 2:** Associations between artificially-sweetened non-carbonated soft drinks consumption during pregnancy and child asthma in the Danish National Birth Cohort.

Frequencyof intake		Cases/N	Asthma (18 months)	*P* for trend**	Cases/N	Asthma (7years - ISAAC)	*P* for trend**	Cases/N	Ever asthma (DNPR)	*P* for trend**	Cases/N	Ever asthma (RMPS)	*P* for trend**
			N = 44,650			N = 38,165			N = 38,272			N = 38,272	
			OR (95% CI)			OR (95% CI)			OR (95% CI)			OR (95% CI)	
Never	Crude	4,632/29,392	1.00 (ref.)	<0.0001	1,018/25,508	1.00 (ref.)	0.20	1,443/25,584	1.00 (ref.)	0.001	7,78725,586	1.00 (ref.)	<0.0001
	Adjusted*			<0.0001			0.84			0.08			0.001
<1 serv/week	Crude	606/3,258	1.22 (1.11, 1.34)		97/2,685	0.90 (0.73, 1.12)		172/2,692	1.14 (0.97, 1.34)		851/2,690	1.06 (0.97, 1.15)	
	Adjusted*		1.22 (1.09, 1.36)			0.98 (0.76, 1.27)			1.20 (0.98, 1.48)			1.06 (0.95, 1.18)	
Weekly	Crude	1,179/6,137	1.27 (1.18, 1.36)		216/5,087	1.07 (0.92, 1.24)		326/5,102	1.14 (1.01, 1.29)		1,750/5,102	1.19 (1.12, 1.27)	
	Adjusted*		1.21 (1.11, 1.31)			0.94 (0.77, 1.14)			1.13 (0.97, 1.33)			1.07 (0.99, 1.16)	
> = 1 serv/day	Crude	1,202/5,863	1.38 (1.28, 1.48)		210/4,885	1.08 (0.93, 1.26)		333/4,894	1.22 (1.08, 1.38)		1,765/4,894	1.29 (1.21, 1.38)	
	Adjusted*		1.23 (1.13, 1.33)			1.00 (0.82, 1.22)			1.14 (0.98, 1.34)			1.15 (1.06, 1.25)	

* Adjusted for maternal age, smoking, parity, prepregnancy BMI, physical activity, breastfeeding, socioeconomic position, child sex, maternal history of asthma, maternal history of allergies, paternal history of asthma, paternal history of allergies, and energy (in quintiles).

** Median values (0, 0.5, 3.5, and 7) for each intake group entered as a continuous variable into the model.

ISAAC: International Study of Asthma and Allergies in Childhood.

DNPR: Danish National Patient Registry.

RMPS: Register of Medicinal Products Statistics.

#### Ever asthma and current child asthma at the 7 year follow-up

The prevalence of ever asthma by DNPR was 6% (2,265/38,258) and 32% (12,153/38,258) by RMPS. About 4% (1,536/38,149) of children were classified with current asthma. Both carbonated and non-carbonated artificially-sweetened soft drinks were associated with a DNPR and RMPS diagnosis (**Tables 2** **–** **3**). Mothers who drank at least 1 serving of artificially-sweetened carbonated soft drink serving per day were 1.30 (95% CI: 1.01, 1.66) times more likely to have a child with an asthma diagnosis in the DNPR, and 1.13 (95% CI: 0.98, 1.29) times more likely to have a child RMPS asthma diagnosis. For artificially-sweetened non-carbonated soft drinks these odds ratios were 1.14 (95% CI: 0.98, 1.34) and 1.15 (95% CI: 1.06, 1.25) for a DNPR and RMPS asthma diagnosis respectively. No associations were found between maternal intake of sugar-sweetened beverages and child asthma (**Tables S1–2**). Combining artificially-sweetened beverage intake categories strengthened the associations for RMPS asthma diagnosis only (weekly to > = 1serving/day vs. never to <1serving/week: 1.20, 95% CI: 1.07, 1.35). We further excluded the first 3 years of life for the registry diagnoses and found that results strengthened with the DNPR diagnosis for both artificially-sweetened carbonated (> = 1serving/day vs. never: 1.44, 95% CI: 1.00, 2.09) and non-carbonated (> = 1serving/day vs. never: 1.31, 95% CI: 1.03, 1.66) soft drinks.

**Table 3:** Associations between artificially-sweetened carbonated soft drink consumption during pregnancy and child asthma in the Danish National Birth Cohort.

Frequency of intake		Cases/N	Asthma (18 months)	*P* for trend**	Cases/N	Asthma (7 years - ISAAC)	*P* for trend**	Cases/N	Ever asthma (DNPR)	*P* for trend**	Cases/N	Ever asthma (RMPS)	*P* for trend**
			N = 44,638			N = 38,149			N = 38,258			N = N = 38,258	
			OR (95% CI)			OR (95% CI)			OR (95% CI)			OR (95% CI)	
Never	Crude	4,942/29,896	1.00 (ref.)	<0.0001	1,018/25,661	1.00 (ref.)	0.09	1,509/25,733	1.00 (ref.)	0.04	7,944/25,731	1.00 (ref.)	<0.0001
	Adjusted*			0.14			0.27			0.19			0.01
<1 serv/week	Crude	1,013/5,620	1.11 (1.03, 1.20)		185/4,856	0.96 (0.82, 1.13)		287/4,866	1.01 (0.88, 1.15)		1,606/4,868	1.10 (1.03, 1.18)	
	Adjusted*		1.17 (1.07, 1.27)			0.91 (0.74, 1.12)			1.01 (0.85, 1.19)			1.11 (1.02, 1.20)	
Weekly	Crude	1,327/7,399	1.10 (1.03, 1.18)		267/6,189	1.09 (0.95, 1.25)		351/6,212	0.96 (0.85, 1.08)		2,092/6,211	1.14 (1.07, 1.21)	
	Adjusted*		1.06 (0.98, 1.15)			1.04 (0.87, 1.23)			0.98 (0.84, 1.14)			1.10 (1.02, 1.19)	
> = 1 serv/day	Crude	346/1,723	1.27 (1.12, 1.43)		66/1,443	1.16 (0.90, 1.50)		118/1,447	1.43 (1.17, 1.73)		511/1,448	1.22 (1.09, 1.36)	
	Adjusted*		1.10 (0.95, 1.27)			1.18 (0.87, 1.59)			1.30 (1.01, 1.66)			1.13 (0.98, 1.29)	

* Adjusted for maternal age, smoking, parity, prepregnancy BMI, physical activity, breastfeeding, socioeconomic position, child sex, maternal history of asthma, maternal history of allergies, paternal history of asthma, paternal history of allergies, and energy (in quintiles).

** Median values (0, 0.5, 3.5, and 7) for each intake group entered as a continuous variable into the model.

ISAAC: International Study of Asthma and Allergies in Childhood.

DNPR: Danish National Patient Registry.

RMPS: Register of Medicinal Products Statistics.

#### Ever child allergic rhinitis at the 7 year follow-up

Close to 5% (1,855/37,971) of mothers reported an ever child allergic rhinitis doctor diagnosis on the 7 year questionnaire. Artificially-sweetened carbonated beverages were directly related to ever allergic rhinitis (> = 1serving/day vs. never: 1.31, 95% CI: 0.98, 1.74) (**Table 4**). No associations were found for the other types of beverages (**Tables S3**). Combining the artificially-sweetened beverage intake generated results in between the carbonated and non-carbonated intake categories (> = 1serving/day vs. never: 1.11, 95% CI: 0.86, 1.43).

**Table 4:** Associations between artificially-sweetened carbonated and non-carbonated soft drink consumption during pregnancy and self-reported ever child allergic rhinitis in the Danish National Birth Cohort.

Frequency of intake		Cases/N	Artificially-sweetened carbonated soft drinks	*P* for trend**	Cases/N	Artificially-sweetened non-carbonated soft drinks	*P* for trend**
			N = 37,971			N = 37,984	
			OR (95% CI)			OR (95% CI)	
Never	Crude	1,210/25,539	1.00 (ref.)	0.16	1,248/25,384	1.00 (ref.)	0.59
	Adjusted*			0.01			0.83
<1 serv/week	Crude	251/4,835	1.10 (0.96, 1.27)		128/2,666	0.98 (0.81, 1.18)	
	Adjusted*		0.97 (0.80, 1.16)			0.96 (0.75, 1.21)	
Weekly	Crude	319/6,160	1.10 (0.97, 1.25)		243/5,071	0.97 (0.85, 1.12)	
	Adjusted*		1.15 (0.98, 1.35)			0.86 (0.71, 1.04)	
> = 1 serv/day	Crude	75/1,437	1.11 (0.87, 1.41)		231/4,863	0.97 (0.84, 1.11)	
	Adjusted*		1.31 (0.98, 1.74)			1.03 (0.86, 1.24)	

* Adjusted for maternal age, smoking, parity, prepregnancy BMI, physical activity, breastfeeding, socioeconomic position, child sex, maternal history of asthma, maternal history of allergies, paternal history of asthma, paternal history of allergies, and energy (in quintiles).

** Median values (0, 0.5, 3.5, and 7) for each intake group entered as a continuous variable into the model.

### Sensitivity Analyses

The difference between crude and adjusted effect estimates was primarily accounted for by maternal prepregnancy BMI, socioeconomic position, and breastfeeding duration. However, most examined covariates were weak confounders and rarely accounted for more than 10% change in the effect estimates. We further adjusted the models for other foods and nutrient intake; however, this did not change the results. When sugar- and artificially-sweetened beverages were mutually adjusted for, some associations weakened (e.g. artificially-sweetened carbonated beverages and child asthma at 18 months), while others strengthened (e.g. artificially-sweetened carbonated beverages and ever allergic rhinitis), but the general direction of the associations and the conclusions did not change. The most notable change was a strengthening of the effect estimate for artificially-sweetened carbonated beverages and ever allergic rhinitis (> = 1serving/day vs. never: 1.42, 95% CI: 1.05, 1.91). Further adjustment for other potential confounders of child asthma and allergies (mode of delivery, pets at home, exposure do smoking during infancy, daycare attendance during infancy, and use of antibiotics and paracetamol during pregnancy and child use of antibiotics at 6 months as suggested in a recent review by Nurmatov et al [36]) did not alter the effect estimates. For example, effect estimates for artificially-sweetened carbonated beverages and asthma at 18 months were 1.07 (95% CI: 0.93, 1.24) (vs. 1.10, 95% CI: 0.95, 1.27) and for artificially-sweetened carbonated beverages and asthma at 7 years 1.17 (95% CI: 0.87, 1.59) (vs. 1.18, 95% CI: 0.87, 1.59). This analysis was performed to examine the stability of the estimates but we are cautious about adding these seven covariates, some of which are conditioning on future conditions, since we might be imposing overadjustment and bias in our analyses [37], [38].

Based on a previous study from our cohort [16] where we found a direct association between low-fat yoghurt intake, and asthma and allergic rhinitis, we examined the relation between intake of artificially-sweetened beverages and low-fat yoghurt. The strongest Spearman correlation was with low-fat yoghurt with fruit (r = 0.07–0.09, P<0.0001) for both carbonated and non-carbonated beverages. When we adjusted for low-fat yoghurt with fruit, there was no change in effect estimates.

To exclude the possibility that our results were mediated by preterm birth [30], we restricted the analyses to term births (>37 and < = 42 weeks of gestation), which did not substantially alter the results. The results for artificially-sweetened carbonated beverages and self-reported allergic rhinitis strengthened (> = 1 serving/day vs. never: 1.44, 95% CI: 1.07, 1.94 vs. 1.31, 95% CI: 0.98, 1.74), while the association between artificially-sweetened non-carbonated beverages and asthma diagnosis by DNPR weakened (> = 1 serving/day vs. never: 1.06, 95% CI: 0.89, 1.27 vs. 1.14, 95% CI: 0.98, 1.34).

## Discussion

In our prospective study of the relation between maternal intake of artificially-sweetened beverages during pregnancy and development of child allergic disease, we found that artificially-sweetened beverages moderately increased risk of asthma and allergic rhinitis both in early and later childhood. These results appeared stronger for carbonated beverages. We did not find similar results for sugar-sweetened beverages, suggesting that artificial sweeteners, rather than other additives and aromatic compounds in soft drinks, may play a role in the development of allergic diseases.

Little is currently know about artificial sweeteners in relation to allergic disease development. Artificial sweeteners, and particularly aspartame, have, however, been studied more broadly in the context of inflammation [18]–[20]. One study examined the effect of aspartame on oxidation in mice brain and liver and found an increase in tumour-necrosis factor-α and lipid peroxidation in the brain, while reducing glutathione [18]. Another study in zebra fish found increased infiltration of inflammatory cells and production of radical oxygen species in the liver and brain after exposure to aspartame [19]. In contrast, they did not find anti-inflammatory properties of aspartame, but found that it did reduce mechanical allodynia in arthritic rats [20]. Aspartame has also been implicated in alleviation of autoimmune disease [20], [39]. One early study examined aspartame in relation to allergic activity, specifically the ability of aspartame to generate ‘allergic’ reactions by inducing histamine release from mast cells and basophils by a pharmacological mechanism or by an IgE-dependent process of mast cell activation [21]. The authors found that aspartame did not act as a direct mast cell secretagogue, and did not cause degranulation of cultured mouse mast cells or human basophils *in vitro*. Aspartame did decrease antigen-induced histamine release from cultured mouse mast cells after long-term exposure only. The authors could not fully explain the origin of this effect but speculated that by increasing mast cell proliferation, it made mast cells less responsive to anaphylactic stimulation.

Among human studies, a randomized, double-blind, cross-over trial among patients with a history of headaches did not find any difference in histamine levels according to aspartame exposure [40]. Aspartame has also shown not to cause allergic reaction in adults when compared to placebo in randomized, double-blinded studies administering aspartame doses at one time point [24], [25]. A number of smaller studies have shown improvement in dermatitis with aspartame avoidance [22], [23], [41]. No human study has specifically investigated the relation between artificial sweeteners and the prevention of allergic disease; and it is unclear whether results from animal experimental and *in vitro* studies can be transposed to the human condition in pregnancy. Furthermore, while majority of these studies involved aspartame; other artificial sweeteners, such as acesulfame-K, sucralose, or saccharine, need to be considered as potential active agents. Soft drinks often contain a mixture of different sweeteners. The predominant sweeteners in soft drinks appearing on the Danish market are aspartame and acesulfame-K [26]. Their average concentration is approximately 2–3-fold higher in carbonated versus noncarbonated soft drinks, which is why we examined them separately. This could explain the slightly stronger effect size we found for carbonated soft drink, and furthermore suggests that less frequently used sweeteners found in non-carbonated beverages, such as cyclamate and saccharine [26], may not be associated with allergic disease outcomes. We were not, however, able to distinguish between intake of specific artificial sweeteners.

It is possible that other additives may account for the observed findings. The role of food additives in allergic disease is well known. However, as these are rare events, establishing a causal link is complex and most of the current knowledge is based on case reports [42]. As food additives may cause allergic reaction, exposing subjects to a group of food additives, including sweeteners, has been recommended in screening for food allergy [43]. The word “food additives” covers a broad range of chemically unrelated substances and this approach highlights the general absence of mechanistic insight for several suspected allergens.

There were several strengths to our study. These included examining detailed dietary data collected in pregnancy in a large, prospective study in relation to several allergic outcomes. We used outcomes both in early and later childhood in order to differentiate potential etiologically pathways between outcomes in infancy that may later resolve and later outcomes that are more likely to capture clinically relevant asthma and allergic rhinitis. Furthermore, we took advantage of both registry-based and self-reported outcome assessment. These allowed us to better identify clinical asthma in the patient registry, particularly after exclusion of early registry diagnoses, an outcome that may not be as well reported by questionnaire. The previous paper comparing different assessment methods of asthma in the DNBC [44] indicated higher specificities (but lower sensitivities) for the registry based outcomes. If the registries are more likely to capture the true asthma cases, while the self-reported outcomes represent a less valid outcome, then this could explain why we observe associations mainly for the registry based outcomes. In further analysis we found that the results strengthened somewhat for ever asthma diagnosed during inpatient visits, suggesting that disease severity may be an important mediating factor. Questionnaires may be better for assessing outcomes such as allergic rhinitis as these are less likely to end up in registries due to more moderate symptom manifestation and more prevalent use of over-the-counter medication. Self-reported asthma may capture mild to moderate disease and can be used for more direct comparison with other studies using the International Study of Asthma and Allergies in Childhood questionnaire.

We found that results differed by outcome with early life outcomes being associated with artificially-sweetened non-carbonated beverages and registry outcomes more strongly associated with higher intake of artificially-sweetened carbonated beverages. This suggests that different sweeteners or other additives may be accounting for some of the relations. Other plausible explanations include a difference in timing of outcome or etiological pathways. For example, the latter may be more governed by T helper (Th) 2 mechanisms while the former by a mixture of Th1- and Th2-driven pathways [45], [46]. As mentioned in the results section, our results strengthened somewhat after we excluded the first 3 years of diagnoses from the hospital registry, influence of artificial sweeteners on pathways in early vs. later childhood may be different. In an attempt to limit residual confounding, in our statistical models we adjusted for numerous covariates covering anthropometry, lifestyle, breastfeeding and familial history of asthma and other known predictors of asthma. However, we may have failed to identify all relevant confounders and thus our findings may be due to residual confounding by unmeasured or unidentified covariates.

Our study was limited primarily by self-reported assessment of diet and outcomes. Although there is always some concern with misclassification when using FFQs for dietary assessment, we expect it to have differentiated well between low and high beverage consumers. We therefore expect any misclassification to have been non-differential and therefore underestimated the true effect estimates. Furthermore, due to the complex mixtures used in different beverages we cannot quantify accurately the amount consumed of individual sweeteners from soft drinks. This may lead to less precise estimation of effect size and contribute to divergent conclusions when comparing the results for carbonated and non-carbonated beverages. Our definition of current asthma has shown high agreement among cases (>60%) and non-cases (>90%) in a recent study from our cohort [44] when compared to the DNPR; yet, the results for the self-reported outcome did not track those of the registry asthma diagnoses. This could have been due to failure to capture clinically-relevant asthma. Finally, potential for selection bias is plausible in a longitudinal study with loss to follow-up. When we compared participants of this study with non-participants, participants tended to display healthier life-style habits. We do however not believe these differences to be large enough to change our findings.

To conclude, our results from a large prospective cohort study are indicative of a weak to moderate association between consumption of artificially-sweetened beverages during pregnancy and offspring risk of asthma and allergic rhinitis. While this could be due to residual confounding, we hypothesize that artificial sweeteners may be the plausible causal agent since no similar associations were found for sugar-sweetened beverages. We do not claim our results to be affirmative, but rather they are suggestive and hypothesis generating. These findings need to be further explored at a mechanistic level and examined with more detailed assessment of exposure to artificial sweeteners.

## Supporting Information

Table S1 **Associations between sugar-sweetened non-carbonated soft drinks consumption during pregnancy and child asthma in the Danish National Birth Cohort.**
(DOCX)

Table S2 **Associations between sugar-sweetened carbonated soft drink consumption during pregnancy and child asthma in the Danish National Birth Cohort.**
(DOCX)

Table S3 **Associations between sugar-sweetened carbonated and non-carbonated soft drinks consumption during pregnancy and self-reported ever child allergic rhinitis in the Danish National Birth Cohort.**
(DOCX)
