# Supplementary material for: Consumption of Artificially-Sweetened Soft Drinks in Pregnancy and Risk of Child Asthma and Allergic Rhinitis
Source: PLoS One. 2013 Feb 27;8(2):e57261. doi: 10.1371/journal.pone.0057261 (PMC3584110; doi:10.1371/journal.pone.0057261)
Supplement: Table S1 — Associations between sugar-sweetened non-carbonated soft drinks consumption during pregnancy and child asthma in the Danish National Birth Cohort. (DOCX) [file pone.0057261.s001.docx]

Table S1. Associations between sugar-sweetened non-carbonated soft drinks consumption during pregnancy and child asthma in the Danish National Birth Cohort

| **Frequency of**  **intake** |  | **Cases/N** | **Asthma**  **(18 months)**  N=44,657  OR (95% CI) | ***P* for trend**  ****** | **Cases/N** | **Asthma**  **(7 years - ISAAC)**  N=38,177  OR (95% CI) | ***P* for trend**** | **Cases/N** | **Ever asthma**  **(DNPR)**  N=38,282  OR (95% CI) | ***P* for trend**** | **Cases/N** | **Ever asthma**  **(RMPS)**  N=38,282  OR (95% CI) | ***P* for trend**  ****** |
| --- | --- | --- | --- | --- | --- | --- | --- | --- | --- | --- | --- | --- | --- |
|  |  |  |  |  |  |  |  |  |  |  |  |  |  |
| Never | Crude  Adjusted* | 2,889/15,818 | 1.00 (ref.) | 0.09  0.85 | 546/13,236 | 1.00 (ref.) | 0.52  0.58 | 828/13,267 | 1.00 (ref.) | 0.58  0.05 | 4,459/13,266 | 1.00 (ref.) | <0.0001  0.07 |
| <1 serv/week | Crude  Adjusted* | 1,021/6,535 | 0.83 (0.77, 0.90)  0.89 (0.81, 0.98) |  | 212/5,603 | 0.91 (0.78, 1.07)  1.03 (0.85, 1.26) |  | 279/5,614 | 0.79 (0.68, 0.90)  0.84 (0.71, 1.01) |  | 1,704/5,616 | 0.86 (0.80, 0.92)  0.94 (0.96, 1.02) |  |
| Weekly | Crude  Adjusted* | 1,997/12,491 | 0.85 (0.80, 0.91)  0.93 (0.86, 1.00) |  | 421/10,843 | 0.94 (0.83, 1.07)  0.97 (0.82, 1.15) |  | 636/10,873 | 0.93 (0.84, 1.04)  0.98 (0.85, 1.12) |  | 3,265/10,870 | 0.85 (0.80, 0.90)  0.93 (0.87, 1.00) |  |
| >=1 serv/day | Crude  Adjusted* | 1,685/9,813 | 0.93 (0.87, 0.99)  0.97 (0.90, 1.06) |  | 362/8,495 | 1.03 (0.90, 1.19)  1.07 (0.90, 1.28) |  | 522/8,528 | 0.98 (0.88, 1.10)  1.12 (0.96, 1.29) |  | 2,709/8,530 | 0.92 (0.87, 0.97)  1.01 (0.94, 1.09) |  |
|  |  |  |  |  |  |  |  |  |  |  |  |  |  |

*Adjusted for maternal age, smoking, parity, prepregnancy BMI, physical activity, breastfeeding, socio-economic status, child sex, maternal history of asthma, maternal history of allergies, paternal history of asthma, paternal history of allergies, and energy (in quintiles).

**Median values (0, 0.5, 3.5, and 7) for each intake group entered as a continuous variable into the model.

ISAAC: International Study of Asthma and Allergies in Childhood

DNPR: Danish National Patient Registry

RMPS: Register of Medicinal Products Statistics
