# Supplementary material for: Consumption of Artificially-Sweetened Soft Drinks in Pregnancy and Risk of Child Asthma and Allergic Rhinitis
Source: PLoS One. 2013 Feb 27;8(2):e57261. doi: 10.1371/journal.pone.0057261 (PMC3584110; doi:10.1371/journal.pone.0057261)
Supplement: Table S2 — Associations between sugar-sweetened carbonated soft drink consumption during pregnancy and child asthma in the Danish National Birth Cohort. (DOCX) [file pone.0057261.s002.docx]

Table S2. Associations between sugar-sweetened carbonated soft drink consumption during pregnancy and child asthma in the Danish National Birth Cohort

| **Frequency of intake** |  | **Cases/N** | **Asthma**  **(18 months)**  N=44,810  OR (95% CI) | ***P* for trend**  ****** | **Cases/N** | **Asthma**  **(7 years - ISAAC)**  N=38,288  OR (95% CI) | ***P* for trend**  ****** | **Cases/N** | **Ever asthma**  **(DNPR)**  N=38,393  OR (95% CI) | ***P* for trend**** | **Cases/N** | **Ever asthma**  **(RMPS)**  N=38,398  OR (95% CI) | ***P* for trend**** |
| --- | --- | --- | --- | --- | --- | --- | --- | --- | --- | --- | --- | --- | --- |
|  |  |  |  |  |  |  |  |  |  |  |  |  |  |
| Never | Crude  Adjusted* | 1,203/7,183 | 1.00 (ref.) | <0.0001  0.11 | 245/6,198 | 1.00 (ref.) | 0.18  0.77 | 383/6,223 | 1.00 (ref.) | 0.26  0.32 | 1,966/6,224 | 1.00 (ref.) | 0.01  0.72 |
|  |  |  |  |  |  |  |  |  |  |  |  |  |  |
| <1 serv/week | Crude  Adjusted* | 1,882/11,875 | 0.94 (0.87, 1.01)  0.98 (0.89, 1.08) |  | 415/10,178 | 1.03 (0.88, 1.21)  1.16 (0.95, 1.43) |  | 577/10,204 | 0.91 (0.80, 1.04)  1.00 (0.85, 1.19) |  | 3,009/10,207 | 0.91 (0.85, 0.97)  0.94 (0.86, 1.03) |  |
|  |  |  |  |  |  |  |  |  |  |  |  |  |  |
| Weekly | Crude  Adjusted* | 3,752/21,582 | 1.05 (0.97, 1.12)  1.05 (0.97, 1.14) |  | 718/18,437 | 0.99 (0.85, 1.14)  1.04 (0.86, 1.26) |  | 1,088/18,489 | 0.95 (0.85, 1.08)  0.97 (0.83, 1.13) |  | 5,924/18,485 | 1.02 (0.96, 1.09)  0.99 (0.91, 1.07) |  |
|  |  |  |  |  |  |  |  |  |  |  |  |  |  |
| >=1 serv/day | Crude  Adjusted* | 813/4,170 | 1.20 (1.09, 1.33)  1.04 (0.92, 1.17) |  | 169/3,475 | 1.24 (1.02, 1.52)  1.20 (0.93, 1.56) |  | 228/3,482 | 1.07 (0.90, 1.27)  0.91 (0.73, 1.14) |  | 1,288/3,482 | 1.27 (1.17, 1.39)  1.07 (0.96, 1.20) |  |
|  |  |  |  |  |  |  |  |  |  |  |  |  |  |

*Adjusted for maternal age, smoking, parity, prepregnancy BMI, physical activity, breastfeeding, socio-economic status, child sex, maternal history of asthma, maternal history of allergies, paternal history of asthma, paternal history of allergies, and energy (in quintiles).

**Median values (0, 0.5, 3.5, and 7) for each intake group entered as a continuous variable into the model.

ISAAC: International Study of Asthma and Allergies in Childhood

DNPR: Danish National Patient Registry

RMPS: Register of Medicinal Products Statistics
