# Supplementary material for: Consumption of Artificially-Sweetened Soft Drinks in Pregnancy and Risk of Child Asthma and Allergic Rhinitis
Source: PLoS One. 2013 Feb 27;8(2):e57261. doi: 10.1371/journal.pone.0057261 (PMC3584110; doi:10.1371/journal.pone.0057261)
Supplement: Table S3 — Associations between sugar-sweetened carbonated and non-carbonated soft drinks consumption during pregnancy and self-reported ever child allergic rhinitis in the Danish National Birth Cohort. (DOCX) [file pone.0057261.s003.docx]

Table S3. Associations between sugar-sweetened carbonated and non-carbonated soft drinks consumption during pregnancy and self-reported ever child allergic rhinitis in the Danish National Birth Cohort

| **Frequency of intake** |  | **Cases/N** | **Carbonated**  **soft drinks**  N=38,111  OR (95% CI) | ***P* for trend**  ****** | **Cases7N** | **Non-carbonated**  **soft drinks**  N=37,996  OR (95% CI) | ***P* for trend**  ****** |
| --- | --- | --- | --- | --- | --- | --- | --- |
|  |  |  |  |  |  |  |  |
| Never | Crude  Adjusted* | 312/6,183 | 1.00 (ref.) | 0.07  0.22 | 669/13,184 | 1.00 (ref.) | 0.68  0.72 |
|  |  |  |  |  |  |  |  |
| <1 serv/week | Crude  Adjusted* | 524/10,134 | 1.03 (0.89, 1.18)  0.96 (0.80, 1.15) |  | 243/5,569 | 0.85 (0.73, 0.99)  0.91 (0.75, 1.10) |  |
|  |  |  |  |  |  |  |  |
| Weekly | Crude  Adjusted* | 857/18,347 | 0.92 (0.81, 1.05)  0.92 (0.78, 1.09) |  | 531/10,797 | 0.97 (0.86, 1.09)  0.94 (0.81, 1.10) |  |
|  |  |  |  |  |  |  |  |
| >=1 serv/day | Crude  Adjusted* | 161/3,451 | 0.92 (0.76, 1.12)  0.86 (0.67, 1.11) |  | 402/8,446 | 0.94 (0.82, 1.06)  1.01 (0.86, 1.20) |  |
|  |  |  |  |  |  |  |  |

*Adjusted for maternal age, smoking, parity, prepregnancy BMI, physical activity, breastfeeding, socio-economic status, child sex, maternal history of asthma, maternal history of allergies, paternal history of asthma, paternal history of allergies, and energy (in quintiles).

**Median values (0, 0.5, 3.5, and 7) for each intake group entered as a continuous variable into the model.
